# Serum YKL-40 Levels in Patients with Asthma or COPD: A Pilot Study

**DOI:** 10.3390/medicina59020383

**Published:** 2023-02-16

**Authors:** Romana Olivia Popețiu, Imola Donath-Miklos, Simona Maria Borta, Silviu Daniel Moldovan, Luminița Pilat, Dragoș Vasile Nica, Maria Pușchiță

**Affiliations:** 1 Department of Internal Medicine, Faculty of Medicine, “Vasile Goldiș” Western University of Arad, 310025 Arad, Romania; 2 Arad County Emergency Clinical Hospital, 310037 Arad, Romania; 3 Department of Physiology, Faculty of Medicine, “Vasile Goldiș” Western University of Arad, 310025 Arad, Romania; 4 Department of Histology, Faculty of Medicine, “Vasile Goldiș” Western University of Arad, 310025 Arad, Romania; 5 Department of Biochemistry, Faculty of Medicine, “Vasile Goldiș” Western Universtiy of Arad, 310025 Arad, Romania; 6 The National Institute of Research—Develpment for Machines and Installations Designed for Agriculture and Food Industry, 077190 Bucuresti, Romania; 7 Research Center for Pharmaco-Toxicological Evaluations, Faculty of Pharmacy, “Victor Babes” University of Medicine and Pharmacy, 300041 Timisoara, Romania

**Keywords:** bronchial asthma, chronic obstructive pulmonary disease, serum YKL-40, fibrinogen

## Abstract

*Background and Objectives:* Bronchial asthma (BA) and chronic obstructive pulmonary disease (COPD) are not only common obstructive respiratory conditions but also major causes of morbidity and mortality worldwide. There is, however, a surprising lack of blood-based biomarkers for separating between these pulmonary disorders. The aim of this study was to assess the practical relevance of using serum YKL-40, single or combined, for this purpose. *Materials and Methods*: Subjects included Romanian patients with BA (*n* = 24) or COPD (*n* = 27). YKL-40, fibrinogen, pre-treatment C-reactive protein (CRP), post-treatment CRP, erythrocyte sedimentation rate, interleukin 6 (IL-6), procalcitonin (PCT), absolute neutrophil count, neutrophil percentage, absolute lymphocyte count, lymphocyte percentage, absolute eosinophil count, and eosinophil percentage were measured and compared between these patients. *Results*: This is the first study investigating the clinical significance of serum YKL-40 in delineating between COPD and BA in Caucasian populations. Only fibrinogen and YKL-40 levels were different between COPD and BA, with the measured values being significantly elevated. These patients exhibited distinct inflammatory profiles. Using the upper quartiles of these variables for the pooled study population (YKL-40: 5100 pg/mL; fibrinogen: 552 mg/dL) as cut-off values, subjects were classified into high or low groups. High YKL-40 adults revealed significantly increased PCT levels. High fibrinogen subjects, by contrast, showed significantly elevated IL-6 concentrations and pre-treatment CRP levels. Low YKL-40 and fibrinogen patients showed the absence of COPD. *Conclusions:* Combined use of serum YKL-40 and fibrinogen may be useful for identifying the absence of COPD.

## 1. Introduction

Chronic obstructive pulmonary disease (COPD) and bronchial asthma (BA) are common obstructive respiratory conditions [1]. COPD is one of the top five leading causes of mortality during the 21st century, especially within the oldest age group. For example, it was the third global cause of death in 2019, with approximately 3.23 million deaths [2]. In contrast, respiratory mortalities associated with BA are seven-fold lower than those reported for COPD [3]. However, the global prevalence of this pulmonary disease has increased by 50% during each of the past four decades, so that it currently affects over 300 million people [4].

Of particular medical concern is severe, life-threatening BA [5]. The risk of developing this form of BA increases by seven percent per year until asthmatic adults reach 45 years [5]. Aging is one of the most important factors mediating its development, progression, and exacerbation [5]. Given the acceleration of global population aging and growing incidence of both COPd and BA, these common respiratory disorders have attracted enhanced research interest from medical and pharmaceutical scientists.

Despite sharing many similarities, BA and COPD display many differences related to etiology, mediators, symptoms, inflammatory cells, consequences, and response to therapy [1]. Thus, BA is caused by inflammation and tightening of the muscles around the small airways after exposure to different environmental factors, including airborne allergens, infections, air pollution, toxins, and weather [5]. Characterized by bronchial hyper-reactivity and airway obstruction, this disease generally starts in childhood, although its adult onset is not uncommon [6]. COPD, by contrast, occurs primarily in middle-aged or older adults, with smoking being linked to nine out of ten cases [2]. This common and preventable chronic respiratory condition affects lung parenchyma and peripheral airways. However, unlike the eosinophilic and CD4-driven inflammation seen in BA, it is associated with CD8- and neutrophilic-triggered inflammation [7].

Growing evidence links chronic respiratory diseases with changes in markers of systemic inflammation, such as fibrinogen, C-reactive protein (CRP), erythrocyte sedimentation rate (ESR), interleukin 6 (IL-6), and procalcitonin (PCT), and blood count parameters, such as absolute neutrophil count (ANC), neutrophil percentage, absolute lymphocyte count (ALC), lymphocyte percentage, absolute eosinophil count (AEC), and eosinophil percentage [8,9,10,11,12]. There is surprisingly little knowledge about the practical relevance of these variables for separating between BA and COPD, although the development of such biomarkers is of great interest for daily clinical practice. A promising biomarker is YKL-40—a plasma glycoprotein secreted by different cell types from normal tissues, e.g., activated macrophages, neutrophils, chondrocytes, and synovial cells [13]. This member of the mammalian chitinase-like family of proteins has no specific function. However, it is pivotal for inflammatory processes, extracellular matrix remodeling, and cancer cell proliferation [14]. Recent data provide support for its role in COPD pathogenesis [14,15,16,17,18] and potential applicability as a biomarker to delineate between BA and COPD [14,19]. The latter results were obtained in Asian populations [14,19] and no information exists for Caucasian populations.

In this context, we hypothesized that there may be some differences between BA and COPD with respect to serum YKL-40 concentrations in Caucasians. Selected blood parameters, including fibrinogen, pre-treatment CRP, post-treatment CRP, ESR, IL-6, PCT, ANC, neutrophil percentage, ALC, lymphocyte percentage, AEC, and eosinophil percentage were also measured. The study population was selected after age and sex matching from a population of middle-aged and young-old adults from the western part of Romania that were diagnosed with either COPD or BA. The results of our study have significant implications for understanding the clinical relevance and applicability YKL-40 and other blood-based biomarkers in the context of chronic obstructive respiratory diseases.

## 2. Materials and Methods

This study was conducted at the Department of Pneumology of the Arad County Clinical Hospital (Arad, Romania) in agreement with the Declaration of Helsinki 1964 and later amendments. It was approved by the Ethics Committees (IECs) of the two institutions involved, that is the aforementioned hospital (approval No. 26/9 October 2019) and the “Vasile Goldiș” Western University of Arad, Romania (approval No. 159/12 December 2019). The patients enrolled in this study were selected from a larger pool of subjects who were hospitalized during the period of one year (from June 2020 to May 2021). All patients or their caregivers received and signed informed consent.

### 2.1. Design

This exploratory pilot study used a retrospective cross-sectional design to evaluate the practical relevance of YKL-40 (and other blood parameters) to delineate between BA subjects and COPD subjects. To the best of our knowledge, such basic information is lacking in Europe and is scarce around the world [18,19,20]. Hence, a group of age- and sex- matched patients was selected in order to obtain evidence-based information about the clinical applicability of these biomarkers.

### 2.2. Participants and Measurements

First, 106 subjects were pre-enrolled on a rolling “first come-first served” criterion if they were newly diagnosed with either BA or COPD or already had this diagnosis. The diagnosis was conducted in accordance with the definitions of the Global Initiative for Asthma (GINA) [21] and the Global Initiative for Chronic Obstructive Lung Disease guidelines [22]. Patients with asthma-COPD overlap syndrome (ACOS) were not included in this trial. The study population was next selected after age and sex matching of subjects from this initial cohort. This enabled us to obtain a homogeneous study group. The present investigation was conducted during the coronavirus (COVID-19) pandemic, hence limiting our access to a larger pool of adults with obstructive respiratory diseases.

Diagnosis was conducted via spirometric assessment, i.e., by performing ventilatory probes for pulmonary function. The recommended therapies were homogeneous within the two groups. Inclusion criteria were aged 18 years or older, signed informed consent, newly diagnosed or known chronic pulmonary disease, presence of a forced expiratory volume variability in spirometric assessment, existence of respiratory symptoms (e.g., wheezing, shortness of breath), and ongoing treatment with bronchodilators, inhaled corticosteroids, and antihistamine drugs (only for BA). Exclusion criteria, in addition to the aforementioned ACOS syndrome, were psychiatric diseases (which may affect free will) and positive testing for COVID-19.

With respect to criteria used to differentiate between BA and COPD, the following clinical features were used to identify COPD subjects: onset after age 40; persistence of symptoms despite treatment; abnormal lung function between symptoms; heavy exposure to risk factors, such as tobacco smoke or biomass fuels; symptoms that worsen slowly over time (i.e., progressive course over years); limited relief from rapid-acting bronchodilator treatment; and severe hyperinflation or other changes on chest X-ray. The criteria used to identify BA patients were as follows: onset before age 20 years; symptoms that vary over time, often limiting activity; a record (e.g., spirometry, peak expiratory flow) of variable airflow limitation; family history of asthma or other allergic condition; lung function that may be normal between symptoms; symptoms that vary either seasonally or from year to year; symptoms that improve spontaneously or have an immediate response to bronchodilator treatment or to inhaled corticosteroids (ICS) over a period of weeks; and normal chest X-ray.

Blood samples collected from the study population during the initial consultation (first visit) were used to determine fibrinogen, pre-treatment CRP, ESR, IL-6, PCT, ANC, neutrophil percentage, ALC, lymphocyte percentage, AEC, and eosinophil percentage. Post-treatment CRP was determined in samples collected at the second visit, at 7–10 days after the initial consultation. This aimed to determine the anti-inflammatory efficacy of the treatment administered. All patients stayed in the hospital between the first and the second visit. Most biochemical analyses were performed at the Arad County Clinical Hospital, except for YKL-40 measurements, which were conducted at the “Vasile Goldiș” Western University of Arad. All analyses were run in triplicate and only the mean values were taken into account.

### 2.3. Statistical Analysis

The homogeneity of Caucasian patients diagnosed with BA or COPD in terms of age and sex was analyzed using a Mann–Whitney U test and a Chi-square (χ^2^) test, respectively [23]. The χ^2^ tests were next applied to determine inter-group differences in the distribution of subjects stratified based on the area of origin (urban vs. rural) and current status of smoking (non-smoker vs. smoker). The measured values for fibrinogen, pre-treatment CRP, post-treatment CRP, ESR, IL-6, procalcitonin, YKL-40, ANC, neutrophil percentage, ALC, lymphocyte percentage, AEC, and eosinophil percentage were compared as described above in the case of age data sets (with Mann–Whitney U tests). After that, within-group correlational analysis (Spearman’s correlations) was run for variables that showed significant inter-group differences to determine the associations between these variables and the other blood-based markers. Their strength was described as follows: very weak, *R_s_* = 0.00–0.19; weak, *R_s_* = 0.20–0.39; moderate, *R_s_* = 0.40–0.59; strong, *R_s_* = 0.60–0.79; and very strong, *R_s_* = 0.80–1.00 [24].

Stratification is common in clinical studies because it reduces the variance of continuous variables. In pilot trials, it is often performed on significant variables to increase the likelihood of detecting meaningful patterns. Partition of the study population into two groups is frequently used in exploratory clinical studies due to small to moderate sample size (24–50 participants) [25]. This sample size is recommended to be used for achieving a reliable sample size calculation for future large studies [25]. Selection of an optimal cut-off value in such trials is, however, challenging, especially when dealing with novel (candidate) biomarkers, such as YKL-40. These biomarkers do not have a well-defined reference range of values associated with being healthy or with different diseases [14,17,18,19]. In this case, it is therefore difficult to set a predefined (a priori) threshold value.

YKL-40 was the primary candidate biomarker investigated in this trial. If the measured values for this variable revealed significant inter-group differences, the subjects were classified into two groups based on a threshold defined a posteriori. Literature data indicate that median values of YKL-40 in COPD are significantly higher than in BA, e.g., mild to moderate asthma, 33.3 (22.9–43.5) ng/mL; severe asthma, 43.3 (31.1–75.9) ng/mL; and COPD, 64.0 (37.4–142.2) ng/mL [17]. However, many patients with BA show serum YKL-40 concentrations overlapping with those measured in COPD subjects [14,17,19]. Hence, we chose to select a cut-off value based on the upper quartile, not on the median value. This aimed to increase the likelihood of using YKL-40 to delineate between these categories of patients in a heterogeneous population in terms of COPD and BA phenotypes. In this case (i.e., significant inter-group differences for YKL-40), the other significant variables were stratified using a similar approach. If YKL-40 levels were similar between groups, the study population was divided into two groups using the median values for significant variables as cut-off points.

Differences in the measured values for blood parameters between these strata were analyzed using Mann–Whitney U tests. Finally, the performance of these variables, used as single or combined biomarkers, in separating between BA and COPD were compared. All statistical analyses were performed with the Statistica version 8 software (StatSoft Inc., Tulsa, OK, USA). Statistical significance was defined at a two-tailed *p* value below 0.05.

## 3. Results

### 3.1. Characteristics of the Subjects

After age and sex matching, 24 patients with BA and 27 patients with COPD were selected from the initial pool of 106 pre-enrolled adults (with collected sociodemographic data). Their sociodemographic characteristics are shown in Table 1. The frequency of smokers was more than two-fold higher in COPD than in BA, and this difference was statistically significant (χ^2^ test, *p* = 0.006). However, no differences existed with respect to sex distribution (χ^2^ test, *p* = 0.056) and area of origin (χ^2^ test, *p* = 0.590).

**Figure 1:**
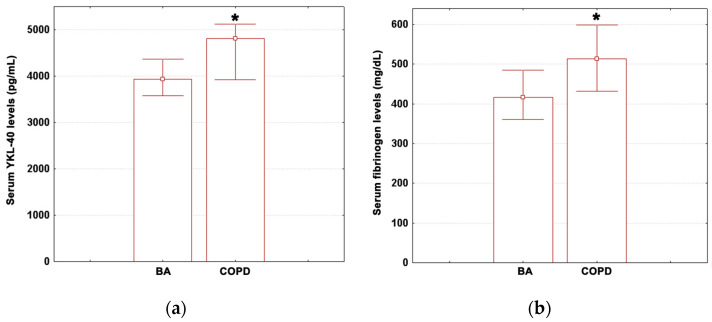
(**a**) YKL-40 levels in COPD and BA; (**b**) fibrinogen concentrations in COPD and BA. Data are shown as median (box) with lower and upper quartiles (error bars). Marked boxes (*) indicate significant differences compared to BA (Mann–Whitney U tests: ***—*p* < 0.001, **—*p* < 0.01, *—*p* < 0.05).

Median values (with lower and upper quartiles) for age, pre-treatment CRP, post-treatment CRP, ESR, IL-6, PCT, ANC, neutrophil percentage, ALC, lymphocyte percentage, AEC, and eosinophil percentage in BA patients and COPD patients are summarized in Table 2. The measured values for YKL-40 and fibrinogen are illustrated in Figure 1a,b. No significant differences in median age were observed between these subjects (Table 2). This attests to the homogeneity of groups in terms of age (Mann–Whitney U test, *p* = 0.821).

Median values for serum YKL-40 concentrations showed a significant 22% increase in COPD as compared to BA (Figure 1a; Mann–Whitney U tests, *p* = 0.038). As a result, the study population was further divided into two groups using the upper quartile of significant variables as cut-off values. Inter-group differences in fibrinogen levels also reached statistical significance (Figure 1b; Mann–Whitney U test, *p* = 0.044), with the median values in COPD patients being 24% above those determined in asthmatic adults. However, no differences were observed for pre-treatment CRP, post-treatment CRP, ESR, IL-6, PCT, ANC, neutrophil percentage, ALC, lymphocyte percentage, AEC, and eosinophil percentage (Table 2; Mann–Whitney U tests, *p* ≥ 0.446).

Serum YKL-40 levels in asthmatic adults showed no significant associations with the other parameters. However, fibrinogen concentrations displayed strong relationships with both pre-treatment CRP (*R_s_* = 0.71) and ESR (*R_s_* = 0.75), but no correlations were found with the other variables. The correlational pattern seen in COPD subjects was different. Thus, fibrinogen correlated significantly only with IL-6, but this correlation was moderate (*R_s_* = 0.47). In contrast, YKL-40 revealed strong associations with neutrophil percentage (*R_s_* = 0.69), lymphocyte percentage (*R_s_* = −0.69), and ALC *(R_s_* = −0.71); moderate associations with PCT *(R_s_* = 0.44) and ANC *(R_s_* = 0.39); but no associations with the other variables.

### 3.2. Variables Associated with High YKL-40 Levels

**Figure 2:**
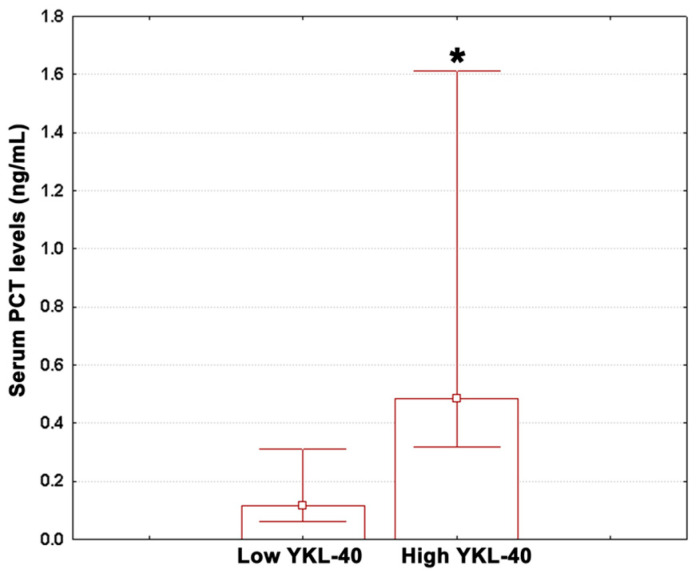
PCT concentrations in low YKL-40 patients and high YKL-40 patients. Data are shown as median (box) with lower and upper quartiles (error bars). Marked boxes (*) indicate significant differences as compared to low YKL-40 subjects (Mann–Whitney U test, ***—*p* < 0.001, **—*p* < 0.01, *—*p* < 0.05).

The study subjects were divided into two groups using the upper quartile of serum YKL-40 of the overall population (5100 pg/mL) as a cut-off point. Median values (with lower and upper quartiles) for fibrinogen, pre-treatment CRP, post-treatment CRP, ESR, IL-6, ANC, neutrophil percentage, ALC, lymphocyte percentage, AEC, and eosinophil percentage in these groups are given in Table 3. The corresponding values for PCT are shown in Figure 2.

PCT levels in subjects from the highest YKL-40 quartile showed a significant three-fold increase relative to low YKL-40 adults (Figure 2; Mann–Whitney U test, *p* = 0.028). In contrast, the measured values for the other variables were similar between groups (Table 3; Mann–Whitney U tests, *p* ≥ 0.088).

### 3.3. Variables Associated with High Fibrinogen Levels

**Figure 3:**
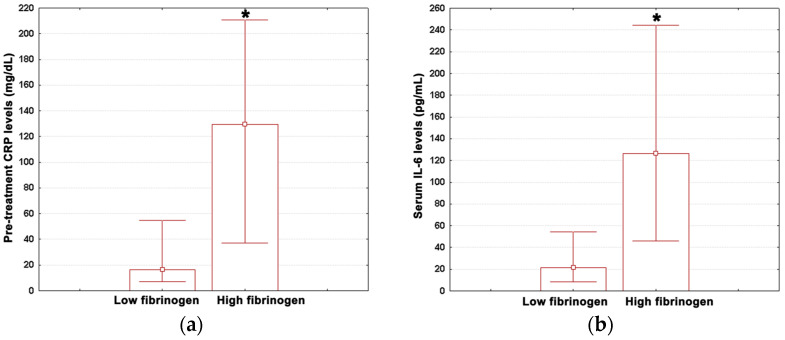
(**a**) Pre-treatment CRP levels in low fibrinogen patients and high fibrinogen patients; (**b**) IL-6 concentrations in high fibrinogen patients and low fibrinogen patients. Data are shown as median (box) with lower and upper quartiles (error bars). Marked boxes (*) indicate significant differences as compared to low fibrinogen adults (Mann–Whitney U test, ***—*p* < 0.001, **—*p* < 0.01, *—*p* < 0.05).

Study patients were classified into two strata using the top quartile for fibrinogen of the overall population as a threshold value (552 mg/dL). Median values (with lower and upper quartiles) for post-treatment CRP, ESR, IL-6, PCT, YKL-40, ANC, neutrophil percentage, ALC, lymphocyte percentage, AEC, and eosinophil percentage are shown in Table 4. The measured values for pre-treatment CRP and IL-6 are given in Figure 3a,b.

High fibrinogen individuals revealed a ten-fold significant increase in pre-treatment CRP levels versus low fibrinogen patients (Figure 3a; Mann–Whitney U test, *p* = 0.017). Similar results were obtained for IL-6 (Figure 3b; Mann-Whitney U test, *p* = 0.013), with the median value being six-fold higher in high fibrinogen adults. In contrast, no significant differences were observed for the other variables analyzed (Table 4; Mann–Whitney U tests, *p* ≥ 0.066).

### 3.4. Performance of Serum YKL-40 and Fibrinogen in Separating BA and COPD

The performance of different partition criteria in separating between BA and COPD is given in Table 5. Using the upper serum YKL-40 quartile as a cut-off point yielded low sensitivity and moderate to high specificity in discriminating between COPD and BA (Table 5). Stratification of patients based on the upper quartile of fibrinogen level had low sensitivity, but high specificity related to this purpose (Table 5). When these two parameters were combined, the sensitivity decreased, but the specificity increased with no patients, with low YKL-40 levels and low fibrinogen concentrations having COPD (Table 5).

## 4. Discussion

The current work provides the first insight into the clinical significance of serum YKL-40 concentrations as a biomarker to distinguish between BA and COPD in Caucasian populations. This expands available knowledge about this topic, which until now was limited to results from Japanese cohorts [14,19]. The results of the present pilot trial are thus of broad interest to the European community of researchers in the field of pulmonary diseases, and especially to those from the Eastern Europe, where the incidence of smoking-associated respiratory disorders is among the highest worldwide [26].

Middle-aged and young-old adults with COPD revealed a significantly higher prevalence of cigarette smoking than those diagnosed with BA. This is in line with the main body of evidence showing that smoking is the strongest risk factor for COPD development [27,28,29]. There is, by contrast, no conclusive indication that smoking causes BA. However, this deleterious behavior can trigger asthmatic attacks and lead to more severe disease over time [27,30].

Serum YKL-40 levels measured in COPD patients were significantly above those seen for BA. These findings complement data from Asian populations [14,19]. However, allele frequencies of polymorphisms of the gene encoding this glycoprotein and other related genes can show significant variations at intra- and inter-populational levels (e.g., Asians vs. Caucasians) [31]. Such caveats yield substantial difficulties in extrapolating results between different populations. Our data hence provide, for the first time, pertinent evidence for the potential clinical applicability of YKL-40 as a biomarker to delineate between COPD and BA in Caucasians.

Both the aforementioned studies on Asian populations did not match patients for age prior to the start of the clinical trial. As a result, the authors conducted statistical age-adjustment to limit the bias of age for cohorts compared. Given the association between blood-based parameters and both age and sex [32], this lack of matching may introduce confounding factors that needs to be controlled for in statistical analysis. In fact, matching for key associated variables is recommended in pilot exploratory studies, like was the case in our work. This enables the use of small and moderate sample sizes by preparing the stratified analysis before the beginning of the study while avoiding the risk of having to run analysis with too many strata but small sample size [33].

Fibrinogen was significantly elevated in COPD relative to BA. El-Shoubargy et al. (2021) investigated the relevance of different blood-based biomarkers for BA and COPD. This study yielded similar results, with the measured values for these respiratory conditions being higher than those determined for healthy controls [34]. Growing evidence supports the existence of a concentration-dependent, significant association between elevated circulating fibrinogen levels and diminished lung function, increased risk of COPD development and exacerbation, and higher declines in FEV_1_/FVC ratio in elderly cohorts [35,36,37,38]. However, there is no consensus about the optimal cut-off for detecting patients at increased risk for COPD exacerbation and death. On the other hand, clinical data provide evidence that a fibrinogen concentration of 350–400 mg/dL can be used as a general threshold for this purpose [37,38,39,40]. Based on these findings, it is obvious that most COPD patients from this trial fall within the high-risk group, and great attention must be paid to their treatment.

Asthmatic patients revealed strong positive associations between fibrinogen and both ESR and pre-treatment CRP, while YKL-40 appeared to be independent of the other variables analyzed. These findings may reflect the well-known fact that fibrinogen, CRP, and ESR are general markers of systemic inflammation and tend to be directly related to each other even in healthy subjects [41,42]. The most significant correlations in COPD patients were, by contrast, found for YKL-40; that is, negative association with lymphocyte-related parameters and positive, but weaker association with neutrophil-related parameters. Given the close connections between YKL-40 and neutrophils, and between neutrophils and lymphocytes [7,13,43], it is plausible that serum YKL-40 elevation mediated by ANC increase yields a decrease in ALC and lymphocyte percentage. This altered balance of neutrophil and lymphocyte levels provides indirect evidence for higher levels of inflammation in COPD than in BA [44].

Interestingly, high YKL-40 subjects exhibited an inflammatory profile characterized by significantly augmented PCT levels. Serving as a marker of bacterial infections, PCT is very useful for guiding antibiotic treatment in respiratory infections [45]. It can be used to predict acute exacerbations of COPD and BA rather than to diagnose or delineate between these pulmonary conditions [46,47,48]. Since this protein is the precursor of calcitonin—a key hormone in calcium homeostasis [49]—these subjects are likely to exhibit altered calcium homeostasis. There is, indeed, growing evidence supporting the role of Ca^2+^ signaling dysfunction in development/exacerbation of chronic respiratory diseases, as recently reviewed [50].

High fibrinogen patients revealed a distinct inflammation pattern compared to high YKL-40 adults, with significantly increased IL-6 and pre-treatment CRP concentrations. IL-6—a key mediator of inflammatory response—induces hepatic synthesis of CRP [51]. This may help explain the simultaneous increase in these biomarkers in subjects from the highest fibrinogen quartile. We note that post-treatment CRP levels in the latter stratum returned to values comparable to those seen in low fibrinogen adults despite being significantly increased pre-treatment. These data confirm the anti-inflammatory efficacy of the treatment administered.

However, there was no relationship between serum YKL-40 and fibrinogen in both BA and COPD. Together with the aforementioned data, our findings suggest that these two variables serve as independent biomarkers. This could reflect different molecular aspects underlying chronic inflammation related to disturbed respiratory function. The use of YKL-40 or fibrinogen as single biomarkers had low sensitivity but relatively high specificity in discriminating between BA and COPD. Their combined use improved specificity, totally separating between these chronic obstructive respiratory conditions. Therefore, patients with low fibrinogen and low YKL-40 levels are very likely to be diagnosed with COPD rather than with BA. Future research hence needs to examine more closely the links between YKL-40 and these diseases. Among other directions for future investigations related to this topic, deciphering the impact of epigenetic changes on phenotypic alterations associated with these lung disorders is of particular interest. Numerous studies have recently emerged with strong indication that epigenetic networks regulating gene expression are pivotal for both BA and COPD development, evolution, and exacerbation [52]. A recent review highlights the potential role of DNA methylation and various post-translational modifications of histones (e.g., acetylation, methylation, and phosphorylation) in modulating gene expression profiles in these diseases [52]. For example, the methylation profiles of *cytosine*-*phosphate*-guanine (*CpG*) sites from the *CHI3L1* gene (encoding YKL-40) can mediate the effect of its genetic variation on serum YKL-40 concentrations in *asthmatics* [53]. Hypermethylation of the *IL-12RBETA2* and *WIF-1* genes can predict COPD evolution towards to lung cancer [54]. Therefore, such epigenetic biomarkers have a broad research prospect in COPD and BA.

The generalizability of the current results is subject to several limitations. With a moderate sample size caution must be applied, as the results might not be transferable to investigations with larger sample sizes. However, such pilot trials are a fundamental stage of clinical research. These studies help clinicians identify and solve critical design issues and evaluate practicality, feasibility, time, resources, and cost of a study before the main research is conducted [33]. Another sources of bias for this investigation may be the use of a cross-sectional design. This type of trial takes place at a specific moment in time and does not follow up individuals over time, and thus cannot provide pertinent information to make a causal inference about the differences observed in the measured values for different blood-based parameters [33]. Other caveats that should be acknowledged are those related to different levels of exposure to environmental or behavioral factors. For example, the study patients were classified as smokers and non-smokers, but we did not collect precise data related to smoking history (ex-smokers were considered as non-smokers) or duration (pack-years), which could possibly interfere with the results obtained [26,27]. Including only hospitalized patients may have also resulted in a bias of selection, since, as it is well known, different phenotypes of asthma and COPD tend to have different rates of exacerbation [14,17,19].

Notwithstanding these limitations, the results of the present study suggest that serum YKL-40 is indeed a promising biomarker for separating between COPD and BA not only in Asians, but also in Caucasians. What are now needed are cross-national studies conducted with larger sample sizes and involving patients with different phenotypes of these diseases. This should help define a more precise reference range of YKL-40 values associated with COPD, BA, and other respiratory diseases. Moreover, having large data sets will facilitate the generation of a receiver operating characteristic curve (ROC) for this biomarker, which, under the present conditions, was not possible due to the exploratory nature of this trial and the use of a unique a posteriori defined cut-off value.

## 5. Conclusions

In conclusion, fibrinogen and serum YKL-40 levels were significantly higher in COPD than in BA. These patients also displayed distinct inflammatory profiles. High YKL-40 adults with either BA or COPD revealed significantly increased PCT levels. In contrast, high fibrinogen subjects showed significantly elevated IL-6 concentrations and pre-treatment CRP levels. The combined use of serum YKL-40 and fibrinogen may be useful for identifying the absence of COPD.

## Figures and Tables

**Table 1:** Sociodemographic characteristics of study patients stratified based on respiratory disease.

	Sex		Smoking Status		Area of Origin	
	Male	Female	Smoker	Non-Smoker	Urban	Rural
BA	7 (38.89%)	11 (61.11%)	7 (38.88%)	11 (61.12%)	6 (16.67%)	12 (83.83%)
COPD	22 (66.66%)	11 (33.34%)	27 (81.81%)	6 (18.19%)	15 (36.36%)	18 (63.64%)

Data are shown as absolute values and the corresponding percentage (in parentheses).

**Table 2:** Measured values for age and selected blood parameters in the study population.

Characteristic	Patients with BA	Patients with COPD
Age (years)	71 (60.5; 74)	68 (64; 74)
Pre-treatment CRP (mg/dL)	14.8 (6.12; 94.84)	36.99 (10; 77)
Post-treatment CRP (mg/dL)	8.64 (3.60; 19.97)	7.42 (2.39; 7.20)
ESR (mm/h)	34 (20.4; 65)	50 (26; 60)
IL-6 (pg/mL)	22.5 (13.33; 79.15)	37.4 (7.43; 74)
PCT (ng/mL)	0.17 (0.06; 0.54)	0.19 (0.07; 0.69)
ANC (10^3^ cells/uL)	7.16 (5.37; 9.98)	7.72 (6.21; 10.45)
Neutrophil percentage (%)	77.10 (54; 84.5)	71.30 (67.30; 81)
ALC (10^3^ cells/uL)	1.61 (0.95; 2.86)	1.49 (1.05; 2.21)
Lymphocyte percentage (%)	13.95 (8.45; 27.45)	16.50 (10.60; 22.70)

Data are shown as median values with lower and upper quartiles (in parentheses). Marked values (*) indicate significant differences as compared to asthmatics (Mann–Whitney U test, ***—*p* < 0.001, **—*p* < 0.01, *—*p* < 0.05).

**Table 3:** Measured values for selected blood parameters in low and high YKL-40 patients.

Characteristic	Low YKL-40 Patients	High YKL-40 Patients
Fibrinogen (mg/dL)	66 (62; 71)	75 (72; 81)
Pre-treatment CRP (mg/dL)	21.06 (10; 75.79)	27.45 (3.52; 204.11)
Post-treatment CRP (mg/dL)	7.42 (3.11; 13.26)	11.30 (2; 28.22)
ESR (mm/h)	50 (26; 60)	39 (23; 66)
IL-6 (pg/mL)	24.9 (12.90; 65.30)	26.47 (9.07; 178)
ANC (10^3^ cells/uL)	7.25 (5.96; 9.59)	10.01 (4.88; 13.04)
Neutrophil percentage (%)	71 (67; 77.90)	82.75 (58.7; 89.40)
ALC (10^3^ cells/uL)	1.90 (1.15; 2.24)	1.25 (0.43; 1.49)
Lymphocyte percentage (%)	17 (12.3; 22.7)	10.05 (3.60; 25.80)
AEC (10^3^ cells/uL)	0.07 (0.02; 0.23)	0.11 (0.03; 0.22)
Eosinophil percentage (%)	0.80 (0.20; 2.10)	1 (0.40; 3.10)

Data are shown as median values with lower and upper quartiles (in parentheses). Marked values (*) indicate significant differences as compared to low YKL-40 patients (Mann–Whitney U tests, ***—*p* < 0.001, **—*p* < 0.01, *—*p* < 0.05).

**Table 4:** Measured values for selected blood parameters in low and high fibrinogen patients.

Characteristic	Low Fibrinogen Patients	High Fibrinogen Patients
Post-treatment CRP (mg/dL)	7.32 (3.11; 15.28)	11.30 (1.52; 28.22)
ESR (mm/h)	44 (23; 58)	60 (42; 64)
PCT (ng/mL)	0.14 (0.06; 0.36)	0.79 (0.07; 1.93))
YKL-40 (pg/mL)	4202 (3914; 4879)	4976 (4511; 7931)
ANC (10^3^ cells/uL)	7.25 (5.79; 9.73)	8.99 (6.91; 10.81)
Neutrophil percentage (%)	70.4 (58.7; 77.9)	77.35 (71.3; 89.4)
ALC (10^3^ cells/uL)	1.72 (1.05; 2.43)	1.26 (0.43; 2.21)
Lymphocyte percentage (%)	17 (10.60; 25.80)	13.35 (3.60; 18)
AEC (10^3^ cells/uL)	0.10 (0.03; 0.23	0.05 (0.034; 0.24)
Eosinophil percentage (%)	0.8 (0.20; 2.80)	0.65 (0.20; 1.40)

Data are shown as median values with lower and upper quartiles (in parentheses). Marked values (*) indicate significant differences compared to low fibrinogen patients (Mann–Whitney U tests, ***—*p* < 0.001, **—*p* < 0.01, *—*p* < 0.05).

**Table 5:** Performance of different stratification criteria in differentiating between BA and COPD.

Stratification Criteria	Sensitivity	Specificity	PPV	NPV
>Q3 YKL-40	29.83%	83.33%	80%	34.48%
>Q3 fibrinogen	33.33%	91.67%	90%	37.93%
>Q3 YKL-40 and >Q3 fibrinogen	14.80%	100%	100%	34.29%

PPV, positive predictive value; NPV, negative predictive value.

## Data Availability

All the data generated or analyzed during this study are included in this published article.

## References

[1] Cukic V., Lovre V., Dragisic D., Ustamujic A. (2012). Asthma and chronic obstructive pulmonary disease (COPD)–differences and similarities. Mater. Sociomed.

[2] Wang G., Ma A., Zhang L., Guo J., Liu Q., Petersen F., Wang Z., Yu X. (2022). Acute exacerbations of chronic obstructive pulmonary disease in a cohort of Chinese never smokers goes along with decreased risks of recurrent acute exacerbation, emphysema and comorbidity of lung cancer as well as decreased levels of circulating eosinophils and basophils. Front. Med..

[3] Gayle A.V., Minelli C., Quint J.K. (2022). Respiratory-related death in individuals with incident asthma and COPD: A competing risk analysis. BMC Pulm. Med..

[4] Song P., Adeloye D., Salim H., Dos Santos J.P., Campbell H., Sheikh A., Rudan I. (2022). Global, regional, and national prevalence of asthma in 2019: A systematic analysis and modelling study. J. Glob. Health.

[5] Zein J.G., Dweik R.A., Comhair S.A., Bleecker E.R., Moore W.C., Peters S.P., Busse W.W., Jarjour N.N., Calhoun W.J., Castro M. (2015). Asthma is more severe in older adults. PLoS ONE.

[6] Borta S.M., Donath-Miklos I., Popetiu R., Nica D.V., Nitusca D., Crisan A., Marian C., Puschita M. (2022). Mannose-binding lectin 2 gene polymorphisms and predisposition to allergic bronchial asthma in a western Romanian children population: An observational study. J. Int. Med. Res..

[7] Buist A.S. (2003). Similarities and differences between asthma and chronic obstructive pulmonary disease: Treatment and early outcomes. Eur. Respir. J..

[8] Rovina N., Koutsoukou A., Koulouris N.G. (2013). Inflammation and immune response in COPD: Where do we stand?. Mediators Inflam..

[9] Maspero J., Adir Y., Al-Ahmad M., Celis-Preciado C.A., Colodenco F.D., Giavina-Bianchi P., Labidi H., Ledanois O., Mahoub B., Perng D.W. (2021). Type 2 inflammation in asthma and other airway diseases. ERJ Open Res..

[10] Liu X., Ge H., Feng X., Hang J., Zhang F., Jin X., Bao H., Zhou M., Han F., Li S. (2020). The combination of hemogram indexes to predict exacerbation in stable chronic obstructive pulmonary disease. Front. Med..

[11] Zhang X.Y., Simpson J.L., Powell H., Yang I.A., Upham J.W., Reynolds P.N., Hodge S., James A.l., Jenkins J., Peteres M.J. (2015). Full blood count parameters for the detection of asthma inflammatory phenotypes. Clin. Exp. Allergy.

[12] Solomon Y., Woldu B., Mesfin N., Enawgaw B. (2022). Selected hematological abnormalities and their associated factors among asthmatic patients in Northwest Ethiopia: A cross-sectional study. BMC Pulm. Med..

[13] Schoneveld L., Ladang A., Henket M., Frix A.N., Cavalier E., Guiot J. (2021). YKL-40 as a new promising prognostic marker of severity in COVID infection. Crit. Care.

[14] Shirai T., Hirai K., Gon Y., Maruoka S., Mizumura K., Hikichi M., Hashimoto S. (2019). Combined assessment of serum periostin and YKL-40 may identify asthma-COPD overlap. J. Allergy Clin. Immunol. Pract..

[15] Gao J., Iwamoto H., Koskela J., Alenius H., Hattori N., Kohno N., Laitinen T., Mazur W., Pulkinnen V. (2016). Characterization of sputum biomarkers for asthma-COPD overlap syndrome. Int. J. Chron. Obstruct. Pulmon. Dis..

[16] Han S.S., Le W.H., Hong Y., Kim W.J., Yang J.H., Lim M.N., Lee S.J., Kwon J.W. (2016). Comparison of serum biomarkers between patients with asthma and with chronic obstructive pulmonary disease. J. Asthma.

[17] James A.J., Reinius L.E., Verhoek M., Gomes A., Kupczyk M., Hammar U., Ono J., Ohta S., Izuhara K., Bel E. (2016). Increased YKL-40 and chitotriosidase in asthma and chronic obstructive pulmonary disease. Am. J. Respir. Crit. Care Med..

[18] Tong X., Wang D., Liu S., Ma Y., Li Z., Tian P., Fan H. (2018). The YKL-40 protein is a potential biomarker for COPD: A meta-analysis and systematic review. Int. J. Chron. Obstruct. Pulmon. Dis..

[19] Gon Y., Maruoka S., Ito R., Mizumura K., Kozu Y., Hiranuma H., Hattori T., Takahashi M., Hikichi M., Hashimoto S. (2017). Utility of serum YKL-40 levels for identification of patients with asthma and COPD. Allergol. Int..

[20] Peng J., Wang M., Wu Y., Shen Y., Chen L. (2022). Clinical indicators for Asthma-COPD overlap: A systematic review and meta-analysis. Int. J. Chron. Obstruct. Pulmon. Dis..

[21] Global Initiative for Asthma (2022). Global Strategy for Asthma Management and Prevention. www.ginasthma.org.

[22] Global Initiative for Chronic Obstructive Lung Disease (2022). Global Strategy for the Diagnosis, Management, and Prevention of Chronic Obstructive Pulmonary Disease. www.goldcopd.org.

[23] Grelus A., Nica D.V., Miklos I., Belengeanu V., Ioiart I., Popescu C. (2017). Clinical significance of measuring global hydroxymethylation of white blood cell DNA in prostate cancer: Comparison to PSA in a pilot exploratory study. Int. J. Mol. Sci..

[24] Cohen L., Jarvis P., Fowler L. (2013). Practical Statistics for Field Biology.

[25] Sim J., Lewis M. (2012). The size of a pilot study for a clinical trial should be calculated in relation to considerations of precision and efficiency. J. Clin. Epidemiol..

[26] Gallus S., Lugo A., Liu X., Behrakis P., Boffi R., Bosetti C., Carreras G., Chatenoud L., Clancy L., Continente X. (2021). Who smokes in Europe? Data from 12 European countries in the TackSH survey (2017–2018). J. Epidemiol..

[27] Thomson N.C., Chaudhuri R., Livingston E. (2004). Asthma and cigarette smoking. Eur. Respir. J..

[28] Kurashima K., Takaku Y., Ohta C., Takayanagi N., Yanagisawa T., Kanauchi T., Takahashi O. (2017). Smoking history and emphysema in asthma–COPD overlap. Int. J. Chron. Obstruct. Pulmon. Dis..

[29] Aguiar J.A., Tamminga A., Lobb B., Huff R.D., Nguyen J.P., Kim Y., Dvorkin-Gheva A., Doxey A.C., Hirota J.A. (2019). The impact of cigarette smoke exposure, COPD, or asthma status on ABC transporter gene expression in human airway epithelial cells. Asci. Rep..

[30] Polosa R., Thomson N.C. (2013). Smoking and asthma: Dangerous liaisons. Eur. Clin. Respir. J..

[31] Sohn M.H., Lee J.H., Kim K.W., Kim S.W., Lee S.H., Kim K.E., Kim K.H., Lee C.G., Elias J.A., Lee M.G. (2009). Genetic variation in the promoter region of chitinase 3-like 1 is associated with atopy. Am. J. Respir. Crit. Care Med..

[32] Casimir G.J., Lefèvre N., Corazza F., Duchateau J. (2013). Sex and inflammation in respiratory diseases: A clinical viewpoint. Biol. Sex Differ..

[33] Stewart A. (2016). Basic Statistics and Epidemiology: A Practical Guide.

[34] El-Shourbagy M.A., El Bokhary M.S., Kamal H.M. (2021). The use of biomarkers to predict attacks of severe bronchial asthma and chronic obstructive pulmonary disease. J. Environ. Sci. Int..

[35] Zhou B., Liu S., He D., Wang K., Wang Y., Yang T., Zhang Q., Zhang Z., Niu W. (2020). Fibrinogen is a promising biomarker for chronic obstructive pulmonary disease: Evidence from a meta-analysis. Biosci. Rep..

[36] Carriero V., Bertolini F., Sprio A.E., Bullone M., Ciprandi G., Ricciardolo F.L.M. (2020). High levels of plasma fibrinogen could predict frequent asthma exacerbations. J. Allergy Clin. Immunol. Pract..

[37] Valvi D., Mannino D.M., Müllerova H., Tal-Singer R. (2012). Fibrinogen, chronic obstructive pulmonary disease (COPD) and outcomes in two United States cohorts. Int. J. Chron. Obstruct. Pulmon. Dis..

[38] Gava G., Núñez A., Esquinas C., Sarasate M., Loeb E., Pirina P., Miravitlles M., Barrecheguren M. (2020). Analysis of blood biomarkers in patients with Chronic Obstructive Pulmonary Disease (COPD) and with Asthma-COPD Overlap (ACO). COPD J. Chronic Obstr. Pulm. Dis..

[39] Mannino D.M., Tal-Singer R., Lomas D.A., Vestbo J., Barr G., Tetzlaff K., Lowings M., Rennard S.I., Snyder J., Goldman M. (2015). Plasma fibrinogen as a biomarker for mortality and hospitalized exacerbations in people with COPD. Chronic. Obstr. Pulm. Dis..

[40] Mohan M., Parthasarathi A., Chaya S.K., Siddaiah J.B., Mahesh P.A. (2021). Fibrinogen: A feasible biomarker in identifying the severity and acute exacerbation of chronic obstructive pulmonary disease. Cureus.

[41] Bain B.J. (1983). Some influences on the ESR and the fibrinogen level in healthy subjects. Clin. Labor. Haematol..

[42] Qiang F., Xu H., Sheng J. (2022). Relationship between plasma fibrinogen degradation products (FDP) and D-dimer levels and disease activity in rheumatoid arthritis: A STROBE compliant article. Medicine.

[43] Sukrisman L., Sinto R., Priantono D. (2021). Hematologic profiles and correlation between absolute lymphocyte count and neutrophil/lymphocyte ratio with markers of inflammation of COVID-19 in an Indonesian national referral hospital. Int. J. Gen. Med..

[44] Wouters E.F., Reynaert N.L., Dentener M.A., Vernooy J.H. (2009). Systemic and local inflammation in asthma and chronic obstructive pulmonary disease: Is there a connection?. Proc. Am. Thorac. Soc..

[45] Filipsen N., Bro H., Bjerrum L., Jensen J.U.S., Aabenhus R. (2022). The procalcitonin-guided antibiotics in Respiratory Infections (PARI) project in general practice–a study protocol. BMC Prim. Care.

[46] Bafadhel M., Clark T.W., Reid C., Medina M.J., Batham S., Barer M.R., Nicholson K.G., Brightling C.E. (2011). Procalcitonin and C-reactive protein in hospitalized adult patients with community-acquired pneumonia or exacerbation of asthma or COPD. Chest.

[47] Baranov D., Trofimov V. (2022). Procalcitonin as a marker of infectious inflammation in patients with bronchial asthma, COPD, and asthma-COPD overlap. Chest.

[48] Farmani M., Shahini Shams Abadi M., Ahmadi A., Arjmand M.H., Soleimai A., Habibi Dastenaei Z. (2022). Comparing serum procalcitonin levels between patients with acute exacerbation of chronic obstructive pulmonary disease and patients with acute exacerbation of asthma. J. Maz. Univ. Med. Sci..

[49] Sen A., Nigam A., Vachher M. (2022). Role of polypeptide inflammatory biomarkers in the diagnosis and monitoring of COVID-19. Int. J. Pept. Res. Ther..

[50] Shipman J.G., Onyenwoke R.U., Sivaraman V. (2021). Calcium-dependent pulmonary inflammation and pharmacological interventions and mediators. Biology.

[51] Bermudez E.A., Rifai N., Buring J., Manson J.E., Ridker P.M. (2022). Interrelationships among circulating interleukin-6, C-reactive protein, and traditional cardiovascular risk factors in women. Arterioscler. Thromb. Vasc. Biol..

[52] He L.X., Tang Z.H., Huang Q.S., Li W.H. (2020). DNA methylation: A potential biomarker of chronic obstructive pulmonary disease. Front. Cell Dev. Biol..

[53] Guerra S., Melén E., Sunyer J., Xu C.J., Lavi I., Benet M., Bustamante M., Carsin A.E., Dobano C., Guxens M. (2018). Genetic and epigenetic regulation of YKL-40 in childhood. J. Allergy Clin. Immunol..

[54] Suzuki M., Wada H., Yoshino M., Tian L., Shigematsu H., Suzuki H., Alaa M., Tamura H., Fujiwara T., Nagoto K. (2010). Molecular characterization of chronic obstructive pulmonary disease-related non-small cell lung cancer through aberrant methylation and alterations of EGFR signaling. Ann. Surg. Oncol..

